# Successive Site Translocating Inoculation Improved T Cell Responses Elicited by a DNA Vaccine Encoding SARS-CoV-2 S Protein

**DOI:** 10.3389/fimmu.2022.875236

**Published:** 2022-04-19

**Authors:** Xiangxiang Tian, Yifan Zhang, Zhangyufan He, Shaoshuai Li, Dongmei Yan, Zhaoqin Zhu, Yanmin Wan, Wanhai Wang

**Affiliations:** ^1^ Department of Medical Laboratory, First Affiliated Hospital of Zhengzhou University, Zhengzhou, China; ^2^ Department of Infectious Disease, Shanghai Key Laboratory of Infectious Diseases and Biosafety Emergency Response, National Medical Center for Infectious Diseases, Huashan Hospital, Fudan University, Shanghai, China; ^3^ Department of Laboratory Medicine, Shanghai Public Health Clinical Center, Shanghai, China; ^4^ Department of Immunology, School of Basic Medical, Jiamusi University, Jiamusi, China; ^5^ State Key Laboratory of Genetic Engineering, School of Life Science, Fudan University, Shanghai, China; ^6^ Department of Radiology, Shanghai Public Health Clinical Center, Shanghai, China

**Keywords:** successive site translocating inoculation, DNA vaccine, protein vaccine, T cell response, antibody response

## Abstract

A variety of methods have been explored to increase delivery efficiencies for DNA vaccine. However, the immunogenicity of DNA vaccines has not been satisfactorily improved. Unlike most of the previous attempts, we provided evidence suggesting that changing the injection site successively (successively site-translocated inoculation, SSTI) could significantly enhance the immunogenicity of DNA vaccines in a previous study. To simplify the strategy and to evaluate its impact on candidate SARS-CoV-2 vaccines, we immunized mice with either a SARS-CoV-2 spike-based DNA vaccine or a spike protein subunit vaccine *via* three different inoculation strategies. Our data demonstrated that S protein specific antibody responses elicited by the DNA vaccine or the protein subunit vaccine showed no significant difference among different inoculation strategies. Of interest, compared with the conventional site fixed inoculation (SFI), both successive site-translocating inoculation (SSTI) and the simplified translocating inoculation (STI) strategy improved specific T cell responses elicited by the DNA vaccine. More specifically, the SSTI strategy significantly improved both the monofunctional (IFN-γ^+^IL-2^-^TNF-α^-^CD8^+^) and the multifunctional (IFN-γ^+^IL-2^-^TNF-α^+^CD8^+^, IFN-γ^+^IL-2^-^TNF-α^+^CD4^+^, IFN-γ^+^IL-2^+^TNF-α^+^CD4^+^) T cell responses, while the simplified translocating inoculation (STI) strategy significantly improved the multifunctional CD8^+^ (IFN-γ^+^IL-2^-^TNF-α^+^CD8^+^, IFN-γ^+^IL-2^+^TNF-α^+^CD8^+^) and CD4^+^ (IFN-γ^+^IL-2^-^TNF-α^+^CD4^+^, IFN-γ^+^IL-2^+^TNF-α^+^CD4^+^) T cell responses. The current study confirmed that changing the site of intra muscular injection can significantly improve the immunogenicity of DNA vaccines.

## Introduction

According to the data released by WHO, there have been more than 360 million confirmed cases of COVID-19, including over 5.6 million deaths by the end of January 2022. Multiple vaccines have been developed and deployed to reduce severe disease and death, as well as protecting health systems (Interim Statement on COVID-19 vaccines in the context of the circulation of the Omicron SARS-CoV-2 Variant from the WHO TAG-CO-VAC). Both the classical and next-generation platforms have been employed in developing COVID-19 vaccines (1), including whole inactivated virus, live attenuated virus, protein subunit, viral vector, RNA, and DNA vaccines (2, 3). The advantages and disadvantages of these platforms have been described and compared elsewhere (4, 5). Among different types of COVID-19 vaccines, the mRNA vaccines displayed extraordinary performance in preventing COVID-19 according to clinical trials (6, 7). Although there is no parallel comparison of the immunogenicities and protective efficacies, a retrospective review of data generated on rhesus macaque models suggested that mRNA vaccine showed better protection efficacy against SARS-CoV-2 than DNA vaccine (8). Compared with mRNA vaccines, it usually required to use higher dosages of DNA vaccines to stimulate desired immunities (9, 10). A major hurdle that restricts the application of DNA vaccine is the lack of efficient delivery approach (11). A variety of physical methods (gene gun, electroporation, jet injection, etc.), chemical methods (liposome, synthetic polymer, etc.) and adjuvants have been explored to improve the immunogenicity of DNA vaccine (12). However, the extracellular and intracellular barriers that restrain the transportation of DNA into the nucleus have not been satisfactorily resolved (11).

Unlike most of the preceding efforts, in a previous study, we provided the first evidence suggesting that changing the injection site successively (successively site-translocated inoculation, SSTI) could significantly enhance the immunogenicity of a DNA vaccine encoding OVA protein (13). In the current study, we further demonstrate that the SSTI strategy can be leveraged to improve both the quantity and the quality of T cell responses elicited by a candidate DNA vaccine expressing the full length of SARS-CoV-2 S protein. In comparison, the strategy is not conducive to augment the immunogenicity of an alum adjuvanted S protein subunit vaccine.

## Materials and Methods

### Ethics Statement

All experiments and methods were performed in accordance with relevant guidelines and regulations. Experiments using mice were approved by the Research Ethics Review Committee of the Shanghai Public Health Clinical Center Affiliated to Fudan University.

### Construction and Preparation of a Candidate DNA Vaccine Encoding SARS-CoV-2 Full Length S Protein

The full-length s gene sequence of the reference SARS-CoV-2 strain was optimized according to the preference of human codon usage and synthesized by Genewiz (Genewiz Biotech Co., Ltd., Suchow, China). The codon optimized spike gene was subcloned into a eukaryotic expression vector (pJW4303, kindly gifted by Dr. Shan Lu at the University of Massachusetts). And the sequence of inserted gene was verified by Sanger sequencing (Sangon Biotech Co., Ltd., Shanghai, China). An EndoFree plasmid extraction kit (Cat# 12391, Qiagen, Hilden, USA) was used to prepare the recombinant plasmid for mouse vaccination.

### The Preparation of an Alum Adjuvanted Protein Vaccine

Purified SARS-CoV-2 S protein (Cat# 40591-V08H, Sino Biological, China) was reconstituted with PBS at a concentration of 80μg/ml. Then, it was mixed with a commercialized alum adjuvant (Cat# 77161, ThermoFisher Scientific Co., Ltd., USA) at a ratio of 1:1 (v/v). The final concentration of the purified S protein was 40μg/ml.

### Mouse Vaccination

**Figure 1:**
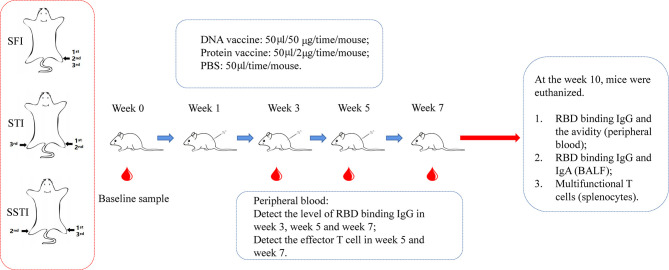
The schematic illustration of experimental design. C57BL/6 mice were immunized with DNA vaccine or alum-adjuvanted protein subunit vaccine *via* 3 different inoculation strategies (SFI, STI and SSTI) for 3 times at an interval of 2 weeks. Peripheral blood samples were collected at baseline and 2 weeks post each immunization. 5 weeks after the last vaccination, mice were euthanized. Mouse serum, splenocytes and BALF were collected for measurements of the antigen-specific immune responses. SFI, site-fixed inoculation; STI, simplified translocating inoculation; SSTI, successively site-translocating inoculation.

Female C57BL/6 mice, 6 to 8 week-old, were housed under a specific-pathogen free (SPF) environment. As shown in **Figure 1**, the mice were immunized intramuscularly with the pJW4303-CMV-S DNA vaccine (50μg/mouse) or the alum adjuvanted S protein subunit vaccine (2μg/mouse) for three times at an interval of 2 weeks. The control group was inoculated with PBS. Peripheral blood was collected at 2 weeks post each vaccination. Five weeks post the third vaccination, the mice were euthanized. Peripheral blood, bronchial alveolar lavage fluid (BALF) and splenocytes were collected for assays of antigen-specific immune responses.

### Detection of SARS-CoV-2 RBD Binding Antibodies

An in-house enzyme-linked immunosorbent assay (ELISA) was developed to measure SARS-CoV-2 RBD specific binding antibody responses. High-binding 96-well EIA plates (Cat# 9018, Corning, USA) were coated with purified SARS-CoV-2 RBD protein (Cat# 40592- V08B, Sino Biological, China) at a final concentration of 1µg/ml in carbonate/bicarbonate coating buffer (30mM NaHCO_3_,10mM Na_2_CO_3_, pH 9.6). Subsequently, the plates were blocked with 1×PBS containing 5% skimmed milk for 1 hour at 37°C. Next, 100μl of serially diluted mouse serum or plasma was added to each well. After 1-hour incubation at 37°C, the plates were washed with 1×PBS containing 0.05% Tween20 for 5 times. Then, 100μl of an HRP labeled goat anti-mouse IgG antibody (Cat# 115-035-003, Jackson Immuno Research, USA) diluted in 1×PBS containing 5% skimmed milk were added to each well and incubated for 1 hour at 37°C. After a second round of wash, 100μl of TMB substrate reagent (Cat# MG882, MESGEN, China) was added to each well. 15 minutes later, the color development was stopped by adding 100μl of 1M H_2_SO_4_ to each well and the values of optical density at OD450_nm_ and OD630_nm_ were measured using 800 TS microplate reader (Cat# 800TS, Biotek, USA). The cut-off value was defined as 2-fold of the average OD450-630 of PBS group at 1:100 dilution.

### Competitive ELISA

The binding antibody titers against the full-length S protein were measured using a method of competitive ELISA (14), which can help to avoid the interference of pre-existing cross-reactive antibody responses against S2. Briefly, high-binding 96-well EIA plates were coated with purified SARS-CoV-2 S protein (Cat# VISC2-S002, East Mab, China) at a final concentration of 1µg/ml in carbonate/bicarbonate coating buffer. The experiment procedure was generally similar with the aforementioned in-house ELISA assays, except that the diluted mouse serum were incubated with a synthesized peptide (P144, SFKEELDKYFKNHT) (10μg/ml) for 1 hour at 37°C before adding into the coated EIA plates.

### Antibody Avidity Assay

Avidity of Ag-specific Ab was determined by avidity ELISA as reported (15–17) with minor modifications. Briefly, plates were coated as the regular ELISA assay described above. Diluted mouse sera were added into each well. After 1-hour incubation, ELISA plates were washed with washing buffer and incubated with 1.5M NaSCN or PBS for 15 minutes at room temperature and then immediately washed with washing buffer. Ab avidity index was defined as the ratio of the OD value of a sample with 1.5M NaSCN treatment versus the OD value of the same sample with PBS treatment.

### Flowcytometry Assays

Freshly isolated splenocytes or peripheral blood mononuclear cells were plated into round-bottom 96-well plates (2×10^6^ cells per well) and incubated with either R10 (RPMI1640 with 10% FBS) or R10 containing synthesized peptides encompassing the full length of S protein (0.66μg/ml for each peptide) (Synthesized by Gill Biochemistry Co., Ltd., Shanghai, China). Two hours later, brefeldin A and monensin were added to each well at final concentrations of 1μg/ml and 1μM, respectively. Another 12 hours later, the cells were washed and stained sequentially with Live/Dead dye (Fixable Viability Stain 510, cat# 564406, BD Pharmingen), surface markers (PE/Cyanine7-labeled anti-mouse CD3, cat# 100220, BioLegend; APC-labeled anti-mouse CD4, cat# 100412, BioLegend; PE-labeled anti-mouse CD8, cat# 100708, BioLegend) and intracellular markers (BV421-labeled anti-mouse IFN-γ, cat# 505830, BioLegend; FITC-labeled anti-mouse IL-2, cat# 503806, BioLegend; BV711-labeled anti-mouse TNF-α, cat# 506349, BioLegend). Stained samples were analyzed using a BD Fortessa flow cytometer and data were analyzed with the FlowJo software version 10 (TreeStar Inc., Ashland, OR, USA). The gating strategy was shown in **Supplementary Figure 1**.

### Statistical Analysis

All statistical analyses were performed using GraphPad Prism 9 (GraphPad Software, Inc., La Jolla, CA, USA). Comparisons between two groups were conducted by the method of t-test. P<0.05 was considered as statistically significant.

## Results

### The S Protein Specific Binding Antibody Responses Were Not Improved by SSTI for Mice Immunized With Both the DNA and Protein Subunit Vaccines

C57BL/6 mice were immunized *via* three different inoculation strategies (**Figure 1**): Mice in the group of site-fixed inoculation (SFI) were immunized by injection into the tibialis anterior of the same limb. Mice in the group of successively site-translocated inoculation (SSTI) were immunized by injecting into the left hind limb, the right hind limb and the left hind limb, sequentially. The mice in the group of simplified translocating inoculation (STI) were injected into the left hind limb for the 1^st^ and 2^nd^ shots, and into the right hind limb for the 3^rd^ shot. Peripheral blood samples were collected at 2 weeks post each immunization. Five weeks post the last vaccination, the mice were euthanized. Serum, BALF and splenocytes were collected for detection of antigen-specific immune responses (**Figure 1**).

**Figure 2:**
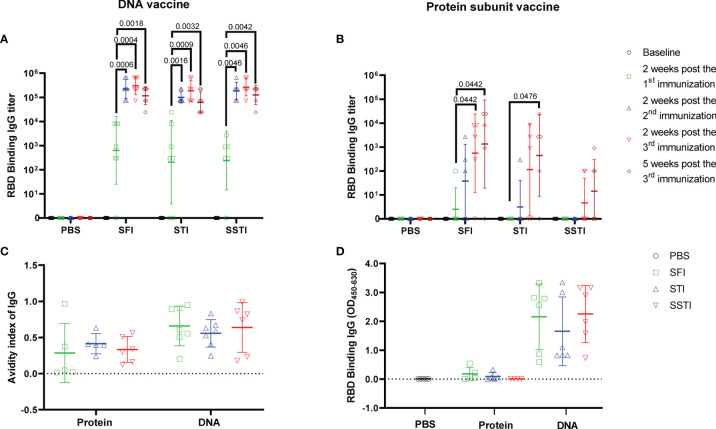
SSTI did not improve the specific antibody responses elicited by the DNA or the protein subunit vaccine. RBD binding antibody titers elicited by the DNA **(A)** or the protein subunit vaccine **(B)** were determined at baseline, 2 weeks post the 1^st^ immunization, 2 weeks post the 2^nd^ immunization, 2 weeks post the 3^rd^ immunization and 5 weeks post the 3^rd^ immunization, respectively. **(C)** The avidity of RBD-specific antibodies was determined at 5 weeks post the 3^rd^ immunization. **(D)** RBD specific IgG in BALF was detected using an ELISA method after adjusting the total protein for each BALF sample to the same concentration (250μg/ml). Data are shown as mean ± SD. Statistical analyses were performed using the method of t-test.

Our data showed that the serum levels of RBD binding IgG titers induced by the DNA and the protein subunit vaccines were not improved by either the SSTI or the STI strategies (**Figures 2A, B**). Of note, the average binding antibody responses elicited by the protein subunit vaccine for the STI and SSTI groups tended to be lower than that of the SFI group (**Figure 2B**). Besides, we found that the binding antibody titers reached the plateau after two shots of DNA vaccine, while it required at least 3 shots for the protein subunit vaccine to boost the binding antibody responses. Similarly, binding antibody titers against the full-length S protein also showed that there was no significant difference among different inoculation strategies (**Supplementary Figure 2**). The avidities of RBD binding antibodies determined at 5 weeks post the 3^rd^ immunization were similar among different strategies (**Figure 2C**). The levels of RBD binding IgG in BALF were different between mice immunized with the DNA vaccine and the protein vaccine. However, no significant difference was observed among different inoculation strategies for either the DNA or the protein subunit vaccine (**Figure 2D**). The levels of RBD binding IgA in BALF were very low in mice inoculated with either the DNA or the protein vaccine (**Supplementary Figure 3**).

### Changing Inoculation Site for the 2^nd^ Shot of DNA Vaccine Was Essential for Eliciting Optimal Specific T Cell Responses

**Figure 3:**
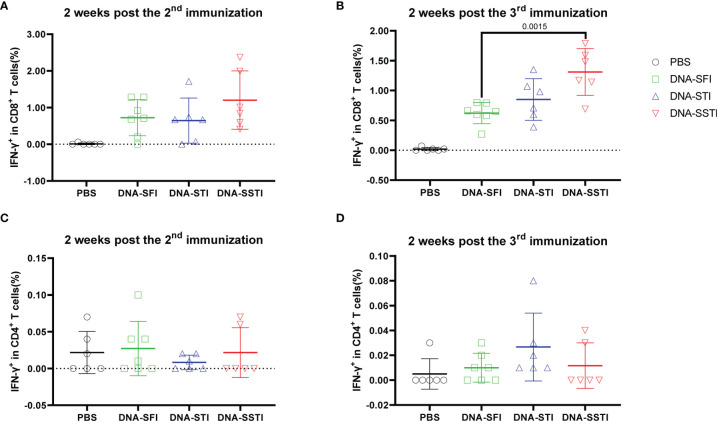
Frequencies of circulating S protein specific IFN-γ secreting CD8^+^ T cells elicited by the DNA vaccine at 2 weeks post the 2^nd^ and 3^rd^ immunization. Peripheral blood were collected from mice immunized with the DNA vaccine at 2 weeks post the 2^nd^ and 3^rd^ immunization. S protein specific IFN-γ secreting CD8^+^ T cells **(A, B)** and CD4^+^ T cells **(C, D)** in peripheral blood were detected by intracellular cytokine staining (ICS). Data are shown as mean ± SD. Statistical analyses were performed using the method of t-test.

**Figure 4:**
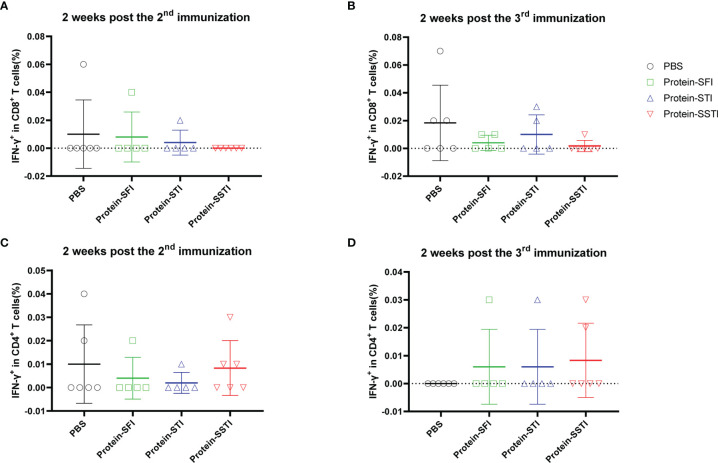
Frequencies of circulating S protein specific IFN-γ secreting CD8^+^ T cells elicited by the protein subunit vaccine at 2 weeks post the 2^nd^ and 3^rd^ immunization. Peripheral blood were collected from mice immunized with the DNA vaccine at 2 weeks post the 2^nd^ and 3^rd^ immunization. S protein specific IFN-γ secreting CD8^+^ T cells **(A, B)** and CD4^+^ T cells **(C, D)** in peripheral blood were detected by intracellular cytokine staining (ICS). Data are shown as mean ± SD. Statistical analyses were performed using the method of t-test.

To characterize the influences of different inoculation strategies on cellular response kinetics during the vaccination process, we collected mouse peripheral blood lymphocytes (PBL) through mandibular vein puncture at 2 weeks post the 2^nd^ immunization and 2 weeks post the 3^rd^ immunization, respectively. S protein specific T cell responses were measured using flowcytometry. Our data showed that the cellular immune responses elicited by the alum adjuvanted S protein were obviously weaker than those induced by the S DNA vaccine (**Figures 3** and **4**). Both the CD8^+^ and CD4^+^ T cell responses at the two time points showed no significant differences among groups of mice immunized with the protein vaccine (**Figure 4**). For mice immunized with the SARS-CoV-2 S DNA vaccine, the average frequency of circulating specific IFN-γ secreting CD8^+^ T cells in the SSTI group (1.310 ± 0.393, 6) was significantly higher than that of the SFI group (0.621 ± 0.177, 7) (P=0.0015) at 2 weeks post the 3^rd^ immunization (**Figure 3B**), whereas the STI strategy did not significantly improve the frequency of circulating IFN-γ^+^CD8^+^ T cells at this time point, suggesting that changing the inoculation site for the second shot of DNA vaccine was essential to induce optimal T cell responses. Interestingly, our data showed that specific CD8^+^ T cell responses elicited by the DNA vaccine were not significantly improved by the SSTI strategy at 2 weeks post the 2^nd^ shot (**Figure 3A**), implying that the SSTI improved DNA induced T cell responses in an incremental mode. Compared with the CD8^+^ T cell responses, the frequencies of S protein specific IFN-γ secreting CD4^+^ T cells in PBL were weak at these time points and showed no significant difference among different inoculation strategies (**Figures 3C, D**).

The incremental improvement of T cell response mediated by the SSTI strategy was verified by an independent experiment using a DNA vaccine encoding a CD8^+^ T cell epitope derived from HIV (18). We used the DNA vaccine immunizing mice *via* two different inoculation strategies: SFI and SSTI (**Supplementary Figure 4A**).Our data showed that antigen-specific CD8^+^ T cell responses in SSTI group were significantly higher than that of the SFI group (P=0.0484) at 2 weeks post the 3^rd^ immunization while not at 2 weeks post the 2^nd^ vaccination (**Supplementary Figure 4B**).

### SSTI Improved the Multifunctional T Cell Responses Induced by the SARS-CoV-2 S DNA Vaccine

**Figure 5:**
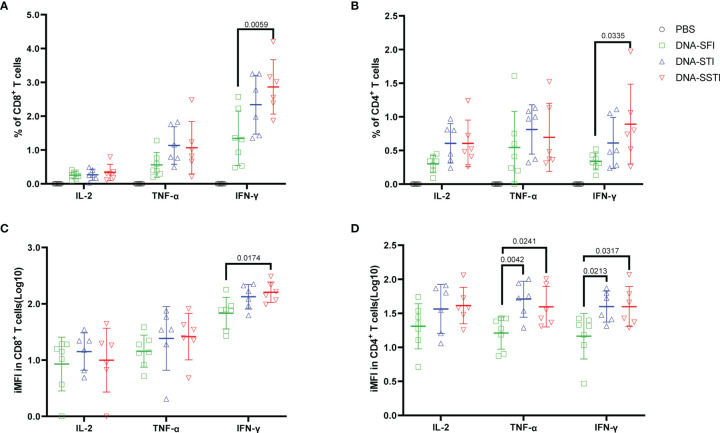
Specific T cell responses elicited by the S DNA vaccine at 5 weeks post the 3^rd^ immunization. Splenocytes were collected from each mouse at 5 weeks post the 3^rd^ immunization. Frequencies of S protein specific IFN-γ, IL-2 and TNF-α secreting CD8^+^ T cells **(A)** and CD4^+^ T cells **(B)** were detected by ICS. iMFI (Specific T cell frequency multiplied by MFI) of specific CD8^+^ **(C)** and CD4^+^ T cells **(D)** was compared among different inoculation strategies. Data are shown as mean ± SD. Statistical differences among groups was analyzed using t-test.

**Figure 6:**
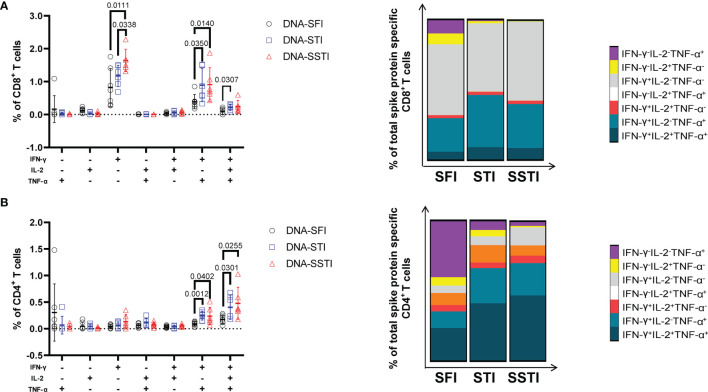
Delineation of multifunctional T cell responses at 5 weeks post 3^rd^ immunization. Multifunctional CD8^+^ **(A)** and CD4^+^ T **(B)** cell responses were analyzed using the Boolean gating strategy (FlowJo). The stacked bar plots depict the proportion of each cell population. Data are shown as mean ± SD. Statistical differences among groups were analyzed using t-test.

To further investigate the influences of different inoculations on the cellular immune responses elicited by the S DNA vaccine, we euthanized the mice at 5 weeks post the 3^rd^ immunization and measured the multifunctionality of S protein specific T cells in spleens using flow cytometry. Compared with the SFI group, the average S protein specific T cell responses (IL-2, TNF-α and IFN-γ) of both the SSTI and the STI groups tended to be higher (**Figures 5A, B**), of which the frequencies of IFN-γ^+^CD8^+^ T cells (**Figure 5A**) and IFN-γ^+^CD4^+^ T cells (**Figure 5B**) were found to be significantly improved in the SSTI group. To evaluate the magnitudes of S protein specific T cells more accurately, we calculated the integrated median fluorescence intensity (iMFI) according to previous reports (18, 19). Our data showed that the iMFI of S protein specific IFN-γ^+^CD8^+^ T cells were significantly improved only by the SSTI strategy (**Figure 5C**). While, the iMFI of TNF-α^+^CD4^+^ cells and IFN-γ^+^CD4^+^ T cells were significantly improved by both the SSTI and STI strategies (**Figure 5D**). Next, the functional features of S protein specific CD8^+^ and CD4^+^ T cells were further delineated *via* Boolean gating. Our data showed that the mean frequency of IFN-γ^+^IL-2^-^TNF-α^-^ CD8^+^ T cells in the SSTI group was significantly higher than those in the STI and the SFI groups (**Figure 6A**). The mean frequencies of IFN-γ^+^IL-2^-^TNF-α^+^CD8^+^ T cells in both the SSTI and the STI groups were significantly higher than that in the SFI group (**Figure 6A**). Similarly, the mean frequencies of IFN-γ^+^IL-2^-^TNF-α^+^ and IFN-γ^+^IL-2^+^TNF-α^+^CD4^+^ T cells were significantly higher in the SSTI and the STI groups than those in the SFI group (**Figure 6B**).

## Disscusion

Compared with conventional vaccine formalities, such as whole inactivated virus vaccine and protein subunit vaccine, an advantage of nucleic acid vaccine is the capability of stimulating stronger antigen specific T cell response (20), which is thought to be important for vaccine mediated protection (21, 22). The contribution of cellular immune response to vaccine-induced protection against COVID-19 has been intensively discussed (23, 24). Its potential to confer cross-protection against different SARS-CoV-2 variants is demonstrated by a few most recent studies (25–27). As a promising nucleic vaccine platform, DNA vaccine has unique advantages such as the low cost and extraordinary stability (28). The major limitation of DNA vaccine is the relatively low immunogenicity compared with mRNA vaccine. Most previous studies seek to improve the immunogenicity of DNA vaccine through optimizing the delivery techniques and/or developing new adjuvants. In a previous study, we showed for the first time that changing the injection site for each shot could significantly improve specific T cell responses elicited by DNA vaccines (13). In this study, we tested whether the SSTI strategy could be leveraged to enhance the cellular immune responses induced by a candidate DNA vaccine encoding SARS-CoV-2 S protein. Meanwhile, we also tried to simplify the SSTI strategy by changing the injection site only for the third (last) shot, which was designated as STI (simplified translocating inoculation).

In consistent with our previous finding (13), we found that neither the SSTI nor the STI strategy significantly improved the SARS-CoV-2 RBD binding antibody responses in mice immunized with 3 shots of a SARS-CoV-2 S DNA vaccine at an interval of 2 weeks. While, the S protein specific CD8^+^ and CD4^+^ T cell responses were significantly improved by the SSTI and the STI strategies at 5 weeks after DNA vaccination. A major difference between SSTI and STI is that STI did not significantly improve circulating CD8^+^ T cell responses at 2 weeks post the 3^rd^ shot, suggesting that the change of inoculation site each time is critical in achieving optimal T cell responses. However, we found that SSTI of two doses didn’t benefit the T cell responses compared with the SFI strategy, which might be because that the local antibody titers induced by the SFI strategy were not high enough to inhibit the expression of DNA vaccine. Indeed, in an on-going mechanism study, we observed that the numbers of residential antibody secreting cell in muscle tissues after 3 times of inoculation were much higher than in those being inoculated only once (Data not shown). Moreover, our data showed that the CD4^+^ and CD8^+^ T cell responses were not equally improved by the SSTI strategy, which might be due to the inequivalent capabilities of the DNA vaccine to induce CD4^+^ and CD8^+^ T cell responses (29). However, the exact mechanism is not clear, which requires further investigation.

In sharp contrast, neither the antibody responses nor the T cell responses elicited by protein vaccine were benefited from the SSTI strategy, which also consisted with our previous finding (13). We have demonstrated in a previous work that ensuring the efficient *in vivo* antigen expression is a major mechanism underlying the SSTI strategy (13).While, the protein-based vaccine does not need to be expressed *in vivo*, therefore, the SSTI strategy doesn`t benefit its immunogenicity. In addition, our data showed that the mucosal IgG tended to be higher in mice inoculated with the DNA vaccine. We speculate that this might be due to the relatively high binding antibody titers in the sera of mice inoculated with the DNA vaccine. It should be noted that the difference observed here may not reflect the real difference between DNA and protein-based vaccines, because the vaccine formulation and dosage were not comparable.

As being discussed elsewhere (30–32), the influence of inoculation route on vaccine immunogenicity has been intensively investigated. In addition to characterizing and comparing the immune responses elicited by different inoculation routes, previous studies also explored to improve topical immune responses *via* combination of different administrating routes (Prime and pull) (33–36). Our current study together with a previous work (13) further show that simply changing the inoculation site for the same route (intra muscular injection) can significantly improve the immunogenicity of DNA vaccines. Since the mechanism of SSTI strategy is different from other immunogenicity enhanced approaches, it is very likely that the strategy can be applied in combination with other techniques, such as gene gun (37) and electroporation (38), which is worth of further investigation. Moreover, as anatomical site-fixed inoculation of any types of vaccine may induce local antibody responses in the injection site, we speculate that the SSTI strategy may also help to improve the immunogenicity of a DNA vaccine even if it is administered as a booster dose.

Several limitations should be noted. First, as aforementioned, our previous study proved that SFI inhibited the *in vivo* antigen expression by local antibodies against the antigen encoded by the DNA vaccine. However, due to technical limitation, we were not able to reliably track and quantify the *in vivo* uptake of plasmid DNA which is a key factor that may impact the *in vivo* expression of DNA vaccine. Second, a challenging experiment with live virus can help to delineate how the enhanced T cell immunities may impact the pathogenecity of SARS-CoV-2. We plan to collaborate with other teams to do the experiment in an ABSL-3 lab in near future. Third, our results were generated using a mouse model, which might not completely mimic the characteristics of human immune responses. We are going to test this strategy through a phase I clinical trial designed to test a DNA based therapeutic TB vaccine in the near future.

## Data Availability Statement

The original contributions presented in the study are included in the article/**Supplementary Material**. Further inquiries can be directed to the corresponding authors.

## Ethics Statement

The animal study was reviewed and approved by Research Ethics Review Committee of the Shanghai Public Health Clinical Center Affiliated to Fudan University.

## Author Contributions

YW and WW designed the study and revised the manuscript. XT, YZ, SL and ZH conducted the experiments and analyzed the data. XT drafted the manuscript. DY and ZZ curated the data and edited the manuscript. All authors listed have made a direct contribution to the work and approved it for publication.

## Funding

This work was partly supported by a grant from the National Natural Science Foundation of China (81971559) and a grant from the major project of Study on Pathogenesis and EpidemicPrevention Technology System (2021YFC2302500) by the Ministry of Science and Technology of China.

## Conflict of Interest

The authors declare that the research was conducted in the absence of any commercial or financial relationships that could be construed as a potential conflict of interest.

## Publisher’s Note

All claims expressed in this article are solely those of the authors and do not necessarily represent those of their affiliated organizations, or those of the publisher, the editors and the reviewers. Any product that may be evaluated in this article, or claim that may be made by its manufacturer, is not guaranteed or endorsed by the publisher.

## References

[1] van Riel D de Wit E. Next-Generation Vaccine Platforms for COVID-19. Nat Mater (2020) 19:810–2. doi: 10.1038/s41563-020-0746-0 32704139

[2] Ndwandwe D Wiysonge CS. COVID-19 Vaccines. Curr Opin Immunol (2021) 71:111–6. doi: 10.1016/j.coi.2021.07.003 PMC8272971 34330017

[3] Nagy A Alhatlani B. An Overview of Current COVID-19 Vaccine Platforms. Comput Struct Biotechnol J (2021) 19:2508–17. doi: 10.1016/j.csbj.2021.04.061 PMC8076774 33936564

[4] Kyriakidis NC Lopez-Cortes A Gonzalez EV Grimaldos AB Prado EO. SARS-CoV-2 Vaccines Strategies: A Comprehensive Review of Phase 3 Candidates. NPJ Vaccines (2021) 6:28. doi: 10.1038/s41541-021-00292-w 33619260 PMC7900244

[5] Versteeg L Almutairi MM Hotez PJ Pollet J. Enlisting the mRNA Vaccine Platform to Combat Parasitic Infections. Vaccines (Basel) (2019) 7:122. doi: 10.3390/vaccines7040122 PMC6963228 31547081

[6] Baden LR El Sahly HM Essink B Kotloff K Frey S Novak R. Efficacy and Safety of the mRNA-1273 SARS-CoV-2 Vaccine. N Engl J Med (2021) 384:403–16. doi: 10.1056/NEJMoa2035389 PMC7787219 33378609

[7] Polack FP Thomas SJ Kitchin N Absalon J Gurtman A Lockhart S. Safety and Efficacy of the BNT162b2 mRNA Covid-19 Vaccine. N Engl J Med (2020) 383:2603–15. doi: 10.1056/NEJMoa2034577 PMC7745181 33301246

[8] Mukhopadhyay L Yadav PD Gupta N Mohandas S Patil DY Shete-Aich A. Comparison of the Immunogenicity & Protective Efficacy of Various SARS-CoV-2 Vaccine Candidates in Non-Human Primates. Indian J Med Res (2021) 153:93–114. doi: 10.4103/ijmr.IJMR_4431_20 33361645 PMC8184077

[9] Liu MA. A Comparison of Plasmid DNA and mRNA as Vaccine Technologies. Vaccines (Basel) (2019) 7:37. doi: 10.3390/vaccines7020037 PMC6631684 31022829

[10] Silveira MM Moreira G Mendonca M. DNA Vaccines Against COVID-19: Perspectives and Challenges. Life Sci (2021) 267:118919. doi: 10.1016/j.lfs.2020.118919 33352173 PMC7749647

[11] Sheridan C. First COVID-19 DNA Vaccine Approved, Others in Hot Pursuit. Nat Biotechnol (2021) 39:1479–82. doi: 10.1038/d41587-021-00023-5 34785814

[12] Eusebio D Neves AR Costa D Biswas S Alves G Cui Z. Methods to Improve the Immunogenicity of Plasmid DNA Vaccines. Drug Discov Today (2021) 26:2575–92. doi: 10.1016/j.drudis.2021.06.008 34214667

[13] Ren Y Wang N Hu W Zhang X Xu J Wan Y. Successive Site Translocating Inoculation Potentiates DNA/recombinant Vaccinia Vaccination. Sci Rep (2015) 5:18099. doi: 10.1038/srep18099 26667202 PMC4678304

[14] Jia L. Pre-Existing Antibodies Targeting a Dominant Linear Antibody Epitope on SARS-CoV-2 S2 Cross-Reacted With Commensal Gut Bacteria and Shaped Immune Responses Elicited by a Candidate Vaccine. medRxiv (2021). doi: 10.1101/2021.07.13.21260404v5

[15] Pratesi F Caruso T Testa D Tarpanelli T Gentili A Gioe D. BNT162b2 mRNA SARS-CoV-2 Vaccine Elicits High Avidity and Neutralizing Antibodies in Healthcare Workers. Vaccines (Basel) (2021) 9:672. doi: 10.3390/vaccines9060672 34207300 PMC8234791

[16] Fan W Wan Y Li Q. Interleukin-21 Enhances the Antibody Avidity Elicited by DNA Prime and MVA Boost Vaccine. Cytokine (2020) 125:154814. doi: 10.1016/j.cyto.2019.154814 31450102

[17] Broni FK Acquah FK Obiri-Yeboah D Obboh EK Sarpong E Amoah LE. Profiling the Quality and Quantity of Naturally Induced Antibody Responses Against Pfs230 and Pfs48/45 Among Non-Febrile Children Living in Southern Ghana: A Longitudinal Study. Front Cell Infect Microbiol (2021) 11:770821. doi: 10.3389/fcimb.2021.770821 34900755 PMC8656302

[18] Wan Y Wang J Zhou H Hu Z Ren X Xu J. The Average IFN-Gamma Secreting Capacity of Specific CD8(+) T Cells Is Compromised While Increasing Copies of a Single T Cell Epitope Encoded by DNA Vaccine. Clin Dev Immunol (2012) 2012:478052. doi: 10.1155/2012/478052 23251217 PMC3509377

[19] Shooshtari P Fortuno ES 3rd Blimkie D Yu M Gupta A Kollmann TR. Correlation Analysis of Intracellular and Secreted Cytokines *via* the Generalized Integrated Mean Fluorescence Intensity. Cytometry A (2010) 77:873–80. doi: 10.1002/cyto.a.20943 PMC2930075 20629196

[20] Qin F Xia F Chen H Cui B Feng Y Zhang P. A Guide to Nucleic Acid Vaccines in the Prevention and Treatment of Infectious Diseases and Cancers: From Basic Principles to Current Applications. Front Cell Dev Biol (2021) 9:633776. doi: 10.3389/fcell.2021.633776 34113610 PMC8185206

[21] Amanna IJ Slifka MK. Contributions of Humoral and Cellular Immunity to Vaccine-Induced Protection in Humans. Virology (2011) 411:206–15. doi: 10.1016/j.virol.2010.12.016 PMC3238379 21216425

[22] World Health Organization. Correlates of Vaccine-Induced Protection: Methods and Implications (2013). Available at: https://www.who.int/publications/i/item/WHO-IVB-13.01 (Accessed May 30, 2013).

[23] Sauer K Harris T. An Effective COVID-19 Vaccine Needs to Engage T Cells. Front Immunol (2020) 11:581807. doi: 10.3389/fimmu.2020.581807 33117391 PMC7549399

[24] Sadarangani M Marchant A Kollmann TR. Immunological Mechanisms of Vaccine-Induced Protection Against COVID-19 in Humans. Nat Rev Immunol (2021) 21:475–84. doi: 10.1038/s41577-021-00578-z PMC8246128 34211186

[25] Tarke A Coelho CH Zhang Z Dan JM Yu ED Methot N. SARS-CoV-2 Vaccination Induces Immunological T Cell Memory Able to Cross-Recognize Variants From Alpha to Omicron. Cell (2022) 185:847–59.ell. doi: 10.1016/j.cell.2022.01.015 PMC8784649 35139340

[26] Kundu R Narean JS Wang L Fenn J Pillay T Fernandez ND. Cross-Reactive Memory T Cells Associate With Protection Against SARS-CoV-2 Infection in COVID-19 Contacts. Nat Commun (2022) 13:80. doi: 10.1038/s41467-021-27674-x 35013199 PMC8748880

[27] Lang-Meli J Luxenburger H Wild K Karl V Oberhardt V Alizei ES. (2022). doi: 10.21203/rs.3.rs-1269004/v1

[28] Khan KH. DNA Vaccines: Roles Against Diseases. Grems (2013) 1:26–35. doi: 10.11599/germs.2013.1034 PMC3882840 24432284

[29] Leifert JA Whitton JL. “Immune Responses to DNA Vaccines: Induction of CD8 T Cells”. In: Madame Curie Bioscience Database. Austin (TX: Landes Bioscience) (2013). p. 2000–13.

[30] Park JH Lee HK. Delivery Routes for COVID-19 Vaccines. Vaccines (Basel) (2021) 9:524. doi: 10.3390/vaccines9050524 34069359 PMC8158705

[31] Mohanan D Slutter B Henriksen-Lacey M Jiskoot W Bouwstra JA Perrie Y. Administration Routes Affect the Quality of Immune Responses: A Cross-Sectional Evaluation of Particulate Antigen-Delivery Systems. J Control Release (2010) 147:342–9. doi: 10.1016/j.jconrel.2010.08.012 20727926

[32] Zhang L Wang W Wang S. Effect of Vaccine Administration Modality on Immunogenicity and Efficacy. Expert Rev Vaccines (2015) 14:1509–23. doi: 10.1586/14760584.2015.1081067 PMC4915566 26313239

[33] Shin H Iwasaki A. A Vaccine Strategy That Protects Against Genital Herpes by Establishing Local Memory T Cells. Nature (2012) 491:463–7. doi: 10.1038/nature11522 PMC3499630 23075848

[34] Roces CB Hussain MT Schmidt ST Christensen D Perrie Y. Investigating Prime-Pull Vaccination Through a Combination of Parenteral Vaccination and Intranasal Boosting. Vaccines (Basel) (2019) 8:10. doi: 10.3390/vaccines8010010 PMC7157738 31906072

[35] Bernstein DI Cardin RD Bravo FJ Awasthi S Lu P Pullum DA. Successful Application of Prime and Pull Strategy for a Therapeutic HSV Vaccine. NPJ Vaccines (2019) 4:33. doi: 10.1038/s41541-019-0129-1 31396405 PMC6671986

[36] Gopinath S Lu P Iwasaki A. Cutting Edge: The Use of Topical Aminoglycosides as an Effective Pull in "Prime and Pull" Vaccine Strategy. J Immunol (2020) 204:1703–7. doi: 10.4049/jimmunol.1900462 PMC11694766 32122994

[37] Bergmann-Leitner ES Leitner WW. Vaccination Using Gene-Gun Technology. Methods Mol Biol (2015) 1325:289–302. doi: 10.1007/978-1-4939-2815-6_22 26450396

[38] Sardesai NY Weiner DB. Electroporation Delivery of DNA Vaccines: Prospects for Success. Curr Opin Immunol (2011) 23:421–9. doi: 10.1016/j.coi.2011.03.008 PMC3109217 21530212

