# Supplementary material for: Successive Site Translocating Inoculation Improved T Cell Responses Elicited by a DNA Vaccine Encoding SARS-CoV-2 S Protein
Source: Front Immunol. 2022 Apr 19;13:875236. doi: 10.3389/fimmu.2022.875236 (PMC9062103; doi:10.3389/fimmu.2022.875236)
Supplement: Supplementary file 1 [file DataSheet_1.docx]

Supplementary Material


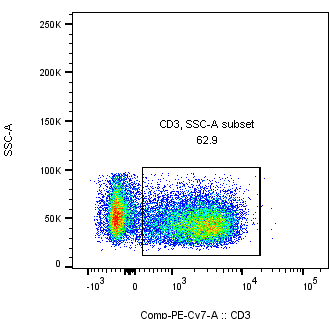

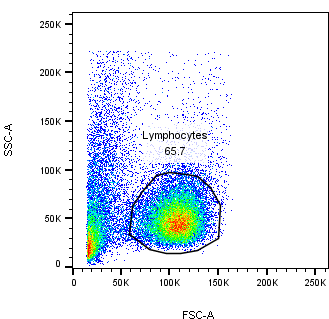

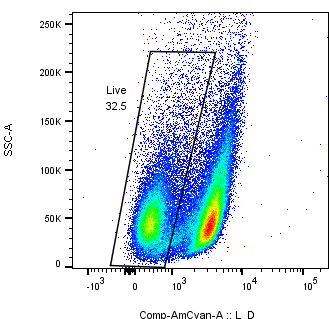

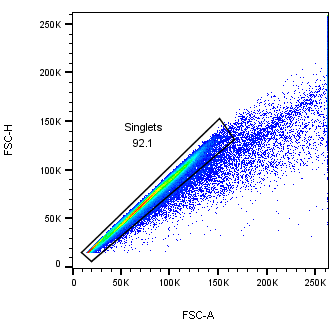

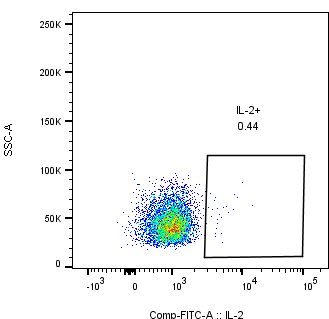

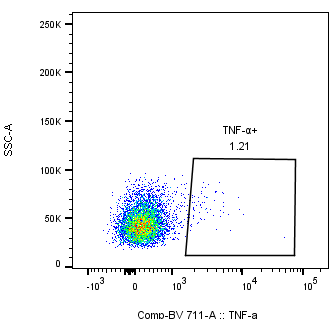

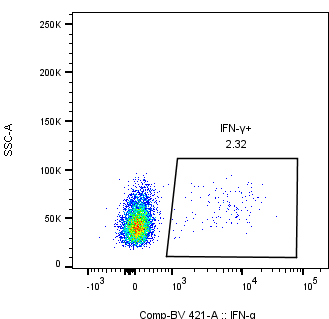

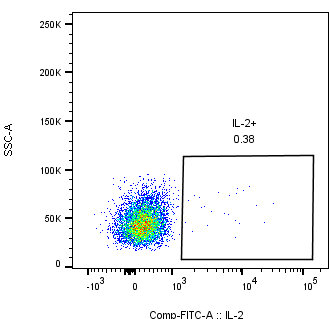

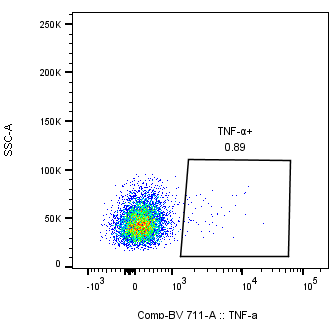

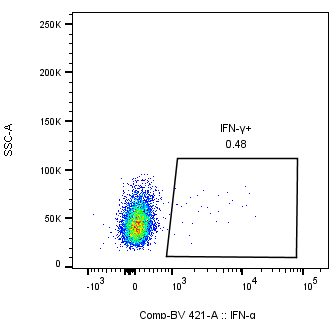

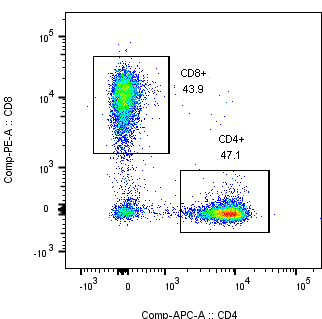


Supplementary Figure 1. Flow cytometry gating strategy. Analyses of polyfunctional T cell responses were done using the method of Boolean Combination Gates (FlowJo).

**Supplementary Figure 2.** S protein binding antibody titers elicited by the DNA or the protein subunit vaccine. S protein binding antibody titers elicited by the DNA (A) or the protein subunit vaccine (B) were determined at baseline, 2 weeks post the 1^st^ immunization, 2 weeks post the 2^nd^ immunization, 2 weeks post the 3^rd^ immunization and 5 weeks post the 3^rd^ immunization, respectively. Data are shown as mean±SD. Statistical analyses were performed using the method of t-test.


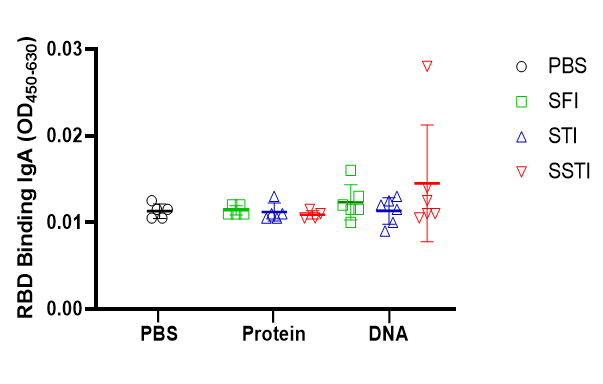


**Supplementary Figure 3.** The RBD binding IgA in BALF. RBD specific IgA in BALF was detected using an ELISA method after adjusting the total protein for each BALF sample to the same concentration (250μg/ml). Data are shown as mean±SD. Statistical analyses were performed using the method of t-test.

**Supplementary Figure 4.** Specific CD8^+^ T cell responses elicited by an epitope-based DNA vaccine were significantly improved by the SSTI strategy after the 3^rd^ immunization while not the 2^nd^ immunization. Peripheral blood were collected from mice immunized with a DNA vaccine encoding a CD8^+^ T cell epitope derived from HIV-1 Env protein at 2 weeks post the 1^st^, 2^nd^ and 3^rd^ immunization, respectively (A). (B) Specific IFN-γ secreting CD8^+^ T cells were detected by intracellular cytokine staining (ICS). Data are shown as mean±SD. Statistical analyses were performed using the method of t-test. SFI: site-fixed inoculation; SSTI: successively site-translocating inoculation.
